# NIDA Clinical Trials Network Common Data Elements Initiative: Advancing Big-Data Addictive-Disorders Research

**DOI:** 10.3389/fpsyt.2015.00033

**Published:** 2015-03-03

**Authors:** Udi E. Ghitza, Robert E. Gore-Langton, Robert Lindblad, Betty Tai

**Affiliations:** ^1^Center for the Clinical Trials Network, National Institute on Drug Abuse, National Institutes of Health, Bethesda, MD, USA; ^2^The EMMES Corporation, Rockville, MD, USA

**Keywords:** substance use disorders, addiction, addiction treatment, drug abuse treatment, health information technology, electronic health record, electronic medical record, medical informatics

## Introduction

The Clinical Trials Network (CTN) of the National Institute on Drug Abuse (NIDA) recently launched a public portal (http://cde.drugabuse.gov) (1), which provides a single-source repository for CTN-recommended common data elements (CDEs) for substance use disorders (SUD) for use in electronic health record systems (EHRs) and clinical research (1, 2). A CDE in this context is a data element consisting of a question and enumerated set of possible values for responses precisely defined by standardized metadata descriptors (1). CDEs consisting of individual question/answer pairs can be combined into more complex questionnaires and case report forms or used when gathering medical information in the context of providing clinical care (1). Thus, CDEs describe semantic characteristics for a discrete piece of data, which will be collected, stored, or exchanged during the course of a study or health examination. This will facilitate exchange of standardized data because of the use of CDEs (1). In this manner, NIDA CDEs can be commonly applied to multiple data collection systems whether in research or clinical care and across different institutions, such that their intentional commonality with use of common data standards can improve data quality, facilitate data re-purposing, and promote data sharing (1, 2). This paper describes objectives and importance of the CTN CDEs initiative and portal to translational psychiatric research: To support harmonized use of EHR-compatible common data elements to enable exchange and integration of data to answer clinically meaningful questions of broad interest to SUD treatment research, thereby facilitating big-data biomedical science crossing boundaries between research and clinical care.

## Approach

Over a series of consensus-building meetings from 2010 to the present, the CTN has led a coordinated effort involving many United States (U.S.) federal government and state agencies, provider organizations, academic institutions, and professional societies to develop CDEs of SUD domains for both EHRs and SUD clinical research (2, 3). The objective of this NIDA-led initiative is to establish measure commonality for SUD care and research that could be integrated into primary care practices in general medical settings, by facilitating the uniform use of EHR-based CDEs (2, 3).

The CTN CDE portal (1) encourages human-subjects research investigators to use, as applicable to their research, a concise set of CDEs when developing case report forms. To ensure meaningful exchange and compatibility of data elements across EHRs and other data management systems, NIDA-recommended CDEs have been developed and curated according to a well-established Metadata Registry standard and archived in the National Cancer Institute’s (NCI) Cancer Data Standards Registry and Repository (caDSR) (1). Definitions and annotations for the NIDA CTN CDEs originate with the NCI Enterprise Vocabulary Service (EVS), which contains standardized terminologies enabling semantic interoperability and health information exchange across EHRs and other data management systems.

One small subset of CDEs is intended for use by practitioners in EHRs of general medical settings, and categorized separately for ease of access (1, 2). EHR vendors are strongly encouraged to select SUD-relevant data elements for their systems from this list, and thus disseminate use of a core set of common standards (2). Additional standardized CDEs are principally intended for clinical research on SUD and co-occurring conditions (e.g., chronic pain, depression), and may also be considered for EHRs for specific purposes (1).

A lack of measure commonality has frequently been encountered in different areas and scientific domains of substance abuse and addiction (SAA) research (4). To address this absence of measure commonality, NIDA strongly encourages NIDA-supported human-subjects research investigators to use Core Tier-1 measures of the SAA project of the phenotypes and exposures (PhenX) Toolkit, as referenced on the NIDA CDE portal. This is a means to facilitate cross-study comparisons and combined data analyses needed to validate and extend human-subjects research results (4). Thus, consistent with NIDA’s efforts to increase measure commonality and establish common data standards across human-subjects research, many of the CTN CDEs in the CTN CDE portal are mapped via CDE entries in the caDSR to Core Tier-1 domains and measures of the SAA PhenX Toolkit (1).

All CTN CDEs are based on a well-established common data standard – the ISO/IEC 11179 metadata registry standard – thus providing semantic interoperability for potential data exchange (1, 2). These CDEs are intended to support useful research using comparative big-data analyses crossing boundaries between human-subjects research and clinical care in large healthcare systems. These are a means by which NIDA advances the U.S. Institute of Medicine’s (IOM’s) vision of a “learning health care system,” in which clinical research could be readily transportable and translatable to practical point-of-care applications at large healthcare system settings and psychiatric practices (5). In addition, integrating CDEs with actionable clinical decision support tools into EHRs of general medical settings may strengthen links between behavioral medicine and primary care in the context of new health reform delivery models, such as Patient-Centered Medical Homes, Accountable Care Organizations, and Coordinated Care Organizations by facilitating standardized systematic data collection, health information exchange, and outcome reporting in these integrated health care systems. Strengthening of care coordination through harmonized data collection and exchange is consistent with one of the pillars of the U.S. Department of Health and Human Services strategic plan on treatment of Multiple Chronic Conditions and for improved quality of patient-centered care (6). It also supports several key attributes defined by the IOM as needed in EHRs functionalities of primary care practices to longitudinally track and provide quality patient-centered care, such as adoption of standardized vocabularies and coding systems (e.g., CDEs) to enable uniform methods for collecting and monitoring patient health information (7).

With increased capacity to share data, safeguarding privacy remains a foremost concern for behavioral health patients and their providers. Title 42, Part 2, of the U.S. Code of Federal Regulations (42 CFR Part 2) is a statute concerning the sharing of substance use information from specific providers. SUD clinical researchers and providers need to safeguard patient privacy in accordance with the confidentiality provisions afforded by 42 CFR Part 2, in addition to those of the Health Insurance Portability and Accountability Act (HIPAA). In addition, in U.S. states where clinical information is collected, there need to be state-wide confidentiality and security regulations and measures in place to specify how protected health information (PHI) data may be accessed, extracted, queried, and analyzed. Furthermore, patients included in clinical research using CDEs need to provide informed consents approved by applicable institutional review boards (IRBs). Also, when clinical-research data are reused, they should be in an aggregated format, and PHI should be excluded to minimize risks of disclosing identities of patients. Previous commentaries have discussed the processes by which meaningful use of EHRs is able to address these considerations and process informed consents and data sharing in accordance with such privacy standards (3, 8).

## Clinical Implications, New Directions, and Conclusion

In sum, NIDA has established expert-defined and consensus-based CDEs (1, 2) housed in the CTN CDE portal (1), which clinical-research investigators and EHR vendors are encouraged to use. Widespread use of these CDEs can accelerate efficient start-up and conduct of new clinical-research projects by providing a set of established data elements from which investigators can select. The vision of the CTN CDEs initiative is to improve quality of data collection in clinical care and biomedical research by fostering standardized utilization of data collection tools that have been validated or vetted by expert groups in the scientific community. Harmonized data collection through use of CTN CDEs can improve big-data biomedical science by facilitating comparison of results across research studies and enable aggregated analyses of data from multiple studies to provide new insight and greater statistical power to answer clinically meaningful questions (2). Through a continued partnership with U.S. federal, state agency, academic institution, provider organization, and professional society stakeholders in updating the CTN CDEs as the science evolves and encouraging their broad use in clinical research and care, NIDA plans to advance biomedical big data accelerating translation of promising research into practical knowledge in a “Learning Health Care system” (5). CDEs crossing boundaries between clinical research and care – such as NIDA CTN CDEs pertinent to clinical researchers, clinicians, and EHR vendors – can help facilitate data integration and reusability across EHRs and clinical data management systems. Thus, they enable transportability of clinical research to healthcare system settings within a “Learning Health Care System” (1, 2, 5).

In order to advance such a system through U.S. Centers for Medicare and Medicaid Services (CMS) quality-improvement reporting and reimbursement programs (9), systematic integration of SUD care into medical settings is needed whereby persons seeking primary care are routinely screened for harmful substance use through validated EHR-based assessment tools (2, 3, 10–12). Importantly, if physicians in general medical settings do not have incentives to screen for harmful drug use and intervene appropriately – guided by pertinent clinical quality measures (CQMs) in EHRs – SUDs would likely go undetected or under-detected in primary care and confound patient-centered care (13). One potential step to promote integration of SUD care with other medical care is inclusion of an EHR-based CQM relevant to substance use screening and intervention for primary-care professionals in CMS-reimbursement programs. Development of an evidence-based CQM reporting on systematic screening, and corresponding interventions, for medically harmful substance use could be used to provide standardized terminology and electronic specification for the uniform collection and exchange of clinically relevant health information in EHRs of integrated healthcare systems. This healthcare delivery integration would in turn help support healthcare program evaluations/quality improvement for treating patients with SUD and chronic co-occurring conditions (13).

To facilitate adoption of validated screening and intervention for SUD in primary care settings – in order to advance systematic collection of biomedical big data in a “Learning Health Care System” crossing boundaries between clinical research and preventive care – the CTN has supported development and electronic specification of an evidence-based composite measure for screening and intervention for substance use (13). The composite CQM is entitled “Substance use screening and intervention composite.” The American Society of Addiction Medicine is the measure steward. Since many patients who are at risk for SUD have not yet developed detectable problems associated with substance use, screening can identify high-risk patients for whom intervention can be indicated. This CQM is defined as the percentage of patients aged 18 years and older who were screened at least once within the last 24 months for tobacco use, unhealthy alcohol use, non-medical prescription drug use, and illicit drug use AND who received an intervention for all positive screening results. Thus, this CQM is intended to evaluate the extent to which primary care patients receive evidence-based screenings for potential abuse of several categories of substances, including tobacco, alcohol, and other drugs, and to encourage comprehensive screening and accompanying intervention (13). A composite CQM was developed to make provider assessments comprehensive. The information in this composite CQM is highly condensed, allowing for the efficient tracking and reporting of a broad range of performance metrics relevant to substance use and abuse. The CTN has provided financial support and oversight for the development and e-specification of this composite CQM with the aim of completing testing of this measure for potential inclusion among CQMs required for eligible professionals in primary care settings to report to obtain CMS-reimbursement incentives (13).

In summary, promoting use of such a CQM and the CDEs in the CTN CDE portal is an important means by which CTN and other stakeholders aim to advance the uniform collection and pooling of big-data sets across studies and sources, including EHRs in general medical settings, to improve data quality and answer clinically meaningful research questions. Using the NIDA CDEs and implementing the aforementioned CQM may be a foundation upon which to develop efficient pragmatic and patient-centered research on how to effectively integrate management of SUD with primary care in general medical settings.

## Conflict of Interest Statement

The authors declare that the research was conducted in the absence of any commercial or financial relationships that could be construed as a potential conflict of interest.
